# Synergistic antibacterial activity of compact silver/magnetite core-shell nanoparticles core shell against Gram-negative foodborne pathogens

**DOI:** 10.3389/fmicb.2022.929491

**Published:** 2022-09-02

**Authors:** Eman M. Sharaf, Amr Hassan, Fawziah A. AL-Salmi, Fauzeya M. Albalwe, Hessa Meteq R. Albalawi, Doaa B. Darwish, Eman Fayad

**Affiliations:** ^1^Department of Bacteriology, Immunology, and Mycology, Animal Health Research Institute (AHRI), Shebin El Kom, Egypt; ^2^Department of Bioinformatics, Genetic Engineering and Biotechnology Research Institute (GEBRI), University of Sadat City, Sadat, Egypt; ^3^Department of Biology, Faculty of Sciences, Taif University, Taif, Saudi Arabia; ^4^Department of Biology, Faculty of Science, Tabuk University, Tabuk, Saudi Arabia; ^5^Department of Pharmacy, Prince Sultan Armed Forces Hospital, Medina, Saudi Arabia; ^6^Department of Botany, Faculty of Science, Mansoura University, Mansoura, Egypt; ^7^Department of Biotechnology, Faculty of Sciences, Taif University, Taif, Saudi Arabia

**Keywords:** foodborne pathogens, silver/magnetite core shell nanoparticle, Gram negative, reactive oxygen species, *Salmonella typhimurium*, *Escherichia coli*, apoptosis

## Abstract

The development of innovative antibacterial drugs against foodborne pathogens has led to an interest in novel materials such as nanomaterials. The unique features of nanomaterial qualify it for use as an antibacterial treatment. Noble metals and metal oxide nanoparticles, such as silver and magnetite nanoparticles, have been shown to be effective antibacterial medications against a range of microorganisms. In this work, Ag@Fe_3_O_4_ -NPs were fabricated by using a wet chemical reduction and modified co-precipitation techniques. The antibacterial efficiency of the Ag/Fe_3_O_4_ core shell nanoparticles was investigated by applying various techniques, such as the Kirby–Bauer Disk Diffusion test, minimum inhibitory concentration (MIC) and bactericidal concentration (MBC), Colony Forming Unit (CFU), and kill time assay. The toxicity mechanism of Ag@Fe_3_O_4_ -NPs against *Salmonella typhimurium* and *Escherichia coli* was studied by apoptosis and reactive oxygen species (ROS) assays. The data revealed that a cubic core was surrounded by a silver shell, which indicated the regular morphology of silver magnetite core shell nanoparticles without any aggregation. Furthermore, Ag@Fe_3_O_4_ -NPs is more toxic against *S. typhimurium* and *E. coli* than Ag-NPs and Fe_3_O_4_ NPs. The MIC values for Ag/Fe_3_O_4_ NPs against *S. typhimurium and E. coli* were 3.1 and 5.4 μg/ml, respectively, whereas the MIC values for Ag-NPs and MNPs against *S. typhimurium* and *E. coli* were 4.1 and 8.2 μg/ml for Ag-NPs and 6.9 and 10.3 μg/ml for MNPs. The results showed the ability of Ag@Fe_3_O_4_ -NPs to induce apoptosis by generating ROS. Also, the ability of Ag@Fe_3_O_4_ -NPs to liberate free Ag^+^ and generate ROS *via* the Haber-Weiss cycle may be a plausible mechanism to explain the toxicity of Ag@Fe_3_O_4_ -NPs - NPs.

## Introduction

Food spoilage is a major health problem in the world due to the presence of foodborne disease germs. Food contamination includes two approaches in the food industry, one of them is linked to environmental conditions such as irrigation water system, infected animals. Whereas, another approach is related to the industrial processes (Scallan et al., 2011; Xu and Sun, 2013). According to the US Centers for Disease Control and Prevention (CDC), *Escherichia coli and Salmonella typhimurium* are most common foodborne diseases, the statistics refer that millions of people in the United States are sicked each year and approximately 450 deaths annually (McNicholl et al., 2007). Antibiotic resistance is a significant problem in the medical section. It has an adverse effect on people’s health. Also, it causes a massive economic loss (Bush et al., 2011). Nowadays, scientists are focusing on developing a novel antibiotic by applying various approaches (Czaplewski et al., 2016; Naylor et al., 2018; Alkawareek et al., 2019). Some bacterial strains formed an adhesion biofilm on synthetic surface which led to form a slime. The biofilm prevented the antibiotic action due to the bacterial resistance (Inbaraj et al., 2012; Dwivedi et al., 2014). Nanomedicine has had a magnificent role in medical applications in the last decade (Brollo et al., 2014). Silver nanoparticles are classified as highly effective bactericides against a broad spectrum of bacteria (Sotiriou and Pratsinis, 2010; Badrelden et al., 2018; Yin et al., 2020). Silver nanoparticles enhance the release of silver ions, which cause the antibacterial activity (Mahdavi et al., 2013). Metal oxide nanoparticles such as titanium dioxide nanoparticles, zinc oxide nanoparticles, and magnetic oxide nanoparticles have antibacterial properties (Hassan et al., 2021). Magnetic nanoparticles (MNPs) also have distinct properties such as biocompatibility, chemical stability, and magnetic properties (Arakha et al., 2015a). By using an external magnetic field, MNPs are applied in molecular target therapy (MTT). Also, MNPs have antibacterial properties due to their ability to produce reactive oxygen species (ROS; Seil and Webster, 2012). Silver magnetic core shell nanoparticles have unique properties such as biocompatibility with chemical stability and higher toxicity against bacteria than Ag-Fe_2_O_3_ heterdimers or plain Ag (Arakha et al., 2015b). Nanosize is characterized by its small size with a high surface area to volume ratio. Hence, the nanoparticle’s high Photoactivity leads to an increase in the production of ROS (Mikhaylova et al., 2004; Garza-Navarro et al., 2010). Recently, scientists have recently become interested in core/shell nanoparticles because of their unique properties and potential applications in a variety of fields, including semiconductor electronic and biomedical applications (Cho et al., 2005), Magnetic resonance imaging for diagnostic and thermal therapies (Cho et al., 2005), drug target therapy (Asmatulu et al., 2005), chemical sensors (Cho et al., 2005), and high-density magnetic recording (Yamaura et al., 2002) are a few examples. Metal-oxide/metal nanoparticles with regulated dimensions are difficult to produce.

Preparation of water-soluble magnetite/silver nanoparticles improves biocompatibility and chemical stability by preventing further oxidation of the magnetic core. In the work, we synthesize silver nanoparticles, magnetite nanoparticles, and silver magnetite core-shell nanoparticles and characterize them all. Then, investigated their antibacterial activity, and the kinetics of inhibitory mechanism against *S. typhimurium* (ATCC14028) and *E. coli* (ATCC35218).

## Materials and methods

### Synthesis and characterization

#### Preparation of silver nanoparticle

Silver nanoparticles (Ag-NPs) were synthesized by a wet chemical reduction approach by using tri-sodium citrate as the reducing and capping agent. In brief, 50 ml of 0.025 mM silver nitrate (AgNO_3_) solution was heated on a hot plate with rapid stirring at 1,400 rpm. Then, 5 ml of tri-sodium citrate (0.02%) was added after the silver nitrate solution reached its boiling point. The reaction was completed after 30 min when the solution color changed to yellow (Algebaly et al., 2020).

#### Preparation of magnetite nanoparticle

According to the Khalafalla and Reimers method, MNPs were synthesized by the addition of ferrous chloride (FeCl_2_.4H_2_O) to ferric chloride (FeCl_3_.6H_2_O) with a molar ratio of (2:3) in 120 ml of milli-Q water (18 ΩM). Finally, 250 ml of NH_4_OH (35 percent V/V) was added and stirred at 1,400 rpm for 30 min. The black precipitate was washed three times with 7% of NH_4_OH, and then the precipitate was collected and dried at room temperature (Khalafalla and Reimers, 1980).

#### Synthesis of silver/magnetite core-shell nanoparticle

Ag@Fe_3_O_4_ -NPs was synthesized by adding colloidal Ag-NPs, which work as a seed for Ag@Fe_3_O_4_ -NPs growth, followed by the addition of magnetite nanoparticles. In brief, 50 ml of silver nanoparticles was added to the magnetite suspension for a half-hour of vigorous stirring at 1,400 rpm. Then, slowly add NH_4_OH (35% V/V) until the color changes to black, then wash many times with milli-Q water (18 ΩM) using an external magnet (Tang et al., 2006).

#### Characterization

The crystalline nature and grain size of the obtained Ag-NPs, MNPs, and Ag@Fe_3_O_4_ -NPs were investigated using XRD patterns at 25–28°C with a D8 Advance X-ray diffractometer (Bruker, Germany). The hydrodynamic size was determined by dynamic light scattering (DLS) and zeta potential measurements (Zetasizer Nano-ZS, Malvern Instruments, United Kingdom). Transmission electron microscopy (TEM, JSM-2100F, JEOL Inc., Tokyo, Japan) and an Atomic Force Microscope (AFM 5600LS, Agilent, United States).

#### Preparation of bacterial strains

Bacterial strains investigated in the current study were *S. typhimurium* ATCC14028 and *E. coli* ATCC35218 were obtained from the American Type Culture Collection (ATCC; Rockville, MD, United States). All the bacterial strains were cultured in Mueller Hinton broth (MHB; Merck, Darmstadt, Germany) at 37°C for 24 h with 200 rpm agitation.

#### Preparation of Resazurin solution

Preparation of Resazurin Solution at 0.03% (w/v) was conducted according to Khalifa et al. (2013). A total of 0.006 g of resazurin salt powder was dissolved in 20 ml of distilled water and stirred and then filtered through a Millipore membrane filter (0.2 mm). The final solution can be kept at 4°C for 14 days.

### *In vitro* susceptibility test

#### Disk diffusion method

The antibacterial activity of Ag-NPs, MNPs, and Ag@Fe_3_O_4_ -NPs against Gram negative foodborne pathogens was investigated using the Kirby–Bauer Disk Diffusion test method (Bauer, 1966). Briefly, Both *S. typhimurium* (ATCC14028) and *E. coli* (ATCC35218) bacteria were spread on Mueller Hinton broth (MHB; Merck, Germany). Following that, paper disks were loaded with 30 μl of all testing samples (colloidal Ag-NPs, MNPs, and Ag@Fe_3_O_4_ -NPs) at a concentration of 10 μg. In addition, 30 μg of Kanamycin and 20 μg of Polymyxin, were used as reference standard antibiotics for *S. typhimurium* and *E. coli*, respectively, which worked as a reference standard antibiotic (positive control), while the non-treated disk served as a negative control, the disks were placed on an agar plate and incubated for 24 h at 37°C. The zone of inhibition was determined after 24 h of incubation.

##### Colony forming unit measurement

Colony Forming Unit (CFU) method counts the number of viable cells for both bacterial strains in the presence of testing materials (colloidal Ag-NPs, MNPs, and Ag@Fe_3_O_4_ -NPs). After 10,000 times dilution in autoclaved deionized water, 10 μl of sample is taken from different concentrations (5, 10, and 25 μg) of colloidal Ag-NPs, MNPs, and Ag@Fe_3_O_4_ -NPs and disseminated on nutrient agar plates. The plates were incubated for 24 h at 37°C. The number of viable cells following exposure to testing material (colloidal Ag-NPs, MNPs, and Ag@Fe_3_O_4_ -NPs) was then determined and compared to a control (non-treated culture) to establish the antibacterial tendency of the studied materials (Seil and Webster, 2012). Additionally, 30 μg of Kanamycin and 20 μg of Polymyxin were added as reference standard antibiotics for *S. typhimurium* and *E. coli*, respectively.

##### Bactericidal concentration and minimum inhibitory concentration assays

Minimum inhibitory concentration (MIC) and bactericidal concentration (MBC) assays were used to evaluate the antimicrobial activity of silver nanoparticles, magnetite nanoparticles, and Ag@Fe_3_O_4_ -NPs (Lau et al., 2014; Loo et al., 2018). The Minimum Inhibitory Concentration assay was carried out in a 96-well round-bottom microtiter plate using the common broth microdilution method, whereas the Minimum Bactericidal Concentration assay was carried out using MHA plates. To initialize, we adjusted the bacterium inoculums to 10^6^ CFU/ml, then added 100 ml of the produced Ag-NPs, MNPs, and Ag@Fe_3_O_4_ -NPs stock solution (500 μM/ml), and diluted 2-fold with the bacterial inoculums in 100 ml of MHB, starting from column 12 to column 3.

The highest concentration of Ag-NPs, MNPs, and Ag@Fe_3_O_4_ -NPs was found in column 12 of the microtiter plate, while the lowest concentration was found in column 3. The first column serves as a negative control (just medium), while the second serves as a positive control (medium and bacterial inoculums). The resazurin solution was then applied to each well of the microtiter plate and incubated for 24 h at 37°C. Bacterial growth was identified by any color shift from blue/purple to pink/colorless. The methodology for determining the MIC value is based on determining the lowest concentrations of Ag-NPs, MNPs, and Ag@Fe_3_O_4_ -NPs that inhibit bacterial growth (color remained blue). The MBC test was carried out by plating the suspension from each well in mircotiter plates on MHA plates and incubating them for 24 h at 37°C. The MBC value was calculated using the lowest concentration with no apparent growth on the MHA plate.

#### Time-kill assay

Killing Kinetic assay was performed in MHB medium, according to Lau et al. (2014) and Zhang et al. (2018). After adjusting the bacterial inoculums to 10^6^ CFU/ml, the testing material solutions (Ag-NPs, MNPs, and Ag@Fe_3_O_4_ -NPs) were diluted with MHB media containing bacterial inoculums to produce the final concentrations of 0x MIC, 1x MIC, 2x MIC, and 4x MIC for each species of bacteria in a total final volume of 1 ml. The cultures were then incubated at 37°C with 150 rpm agitation for 48 h. The cultures were loaded onto MHA plates at different time intervals (0, 30, 60, 120, and 240 min). The experiment was carried out three times. After 24 h of incubation at 37°C, the number of colonies on the MHA plates was counted in CFU/ml.

##### Flow cytometry analysis

*Salmonella typhimurium* and *E. coli* suspension after being inoculated into media containing different concentrations of Ag@Fe_3_O_4_-NPs (0, 5, 10, and 25 μg/L) to investigate apoptosis. After 12 h of incubation, the cultivated cells were collected by centrifugation, washed with 4°C PBS, and centrifuged once more. The supernatant was then discarded, and the cells were resuspended in 300 μl of diluted Binding Buffer, followed by 5 μl of Annexin V-fluorescein isothiocyanate (FITC) and 15 min in the dark. Five minutes before the test, 200 μl of binding buffer was added, along with propidium iodide (PI) dye. The cells were immediately analyzed by flow cytometry (BD FASC Calibur, United States; Chan et al., 2015).

##### Reactive oxygen species

The presence of metal nanoparticles caused the production of ROS, which caused oxidative stress in bacteria. ROS linked with nanoparticles were determined by DCF fluorescence detection. In a nutshell, *S. typhimurium* and *E. coli* suspensions were inoculated in Ag/Fe_3_O_4_ core shell nanoparticles (5, 10, and 25 μg/L) media, and the cells were collected after 12 h and washed three times with PBS buffer. At a volume ratio of 1:2,000, DCFH2-DA and non-treated fresh media were added and incubated at 30°C for 30 min, after which the cells were centrifuged, washed, and resuspended. The cells were immediately analyzed by flow cytometry (BD FASC Calibur, NJ, United States; Chan et al., 2015).

### Statistical analysis

For all experiments, statistical analysis was expressed as mean ± SD of triplicate (independent) experiments. Two sample comparisons of means were carried out using Student’s *t*-test analysis. All analyses were conducted using SPSS 17.0 software (SPSS Inc., Chicago IL, United States). *p* < 0.05 was considered a statistically significant difference.

## Results

### Nanoparticles characterization

**Figure 1:**
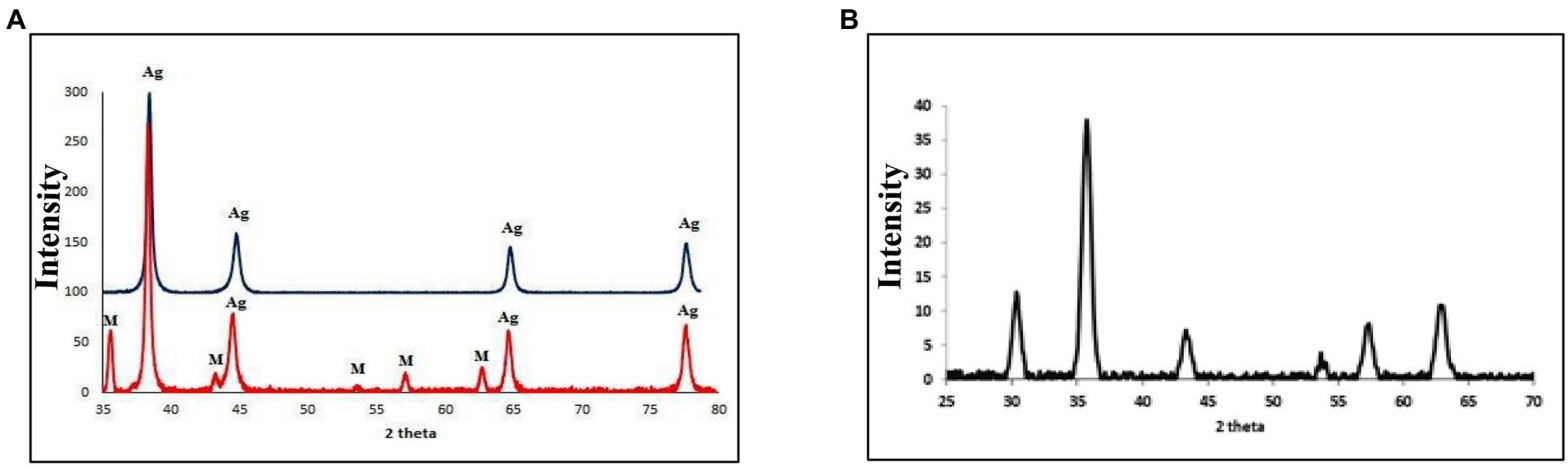
**(A)** X-ray diffraction (XRD) of silver nanoparticles (with blue color), silver/magnetite core shell nanoparticles (with red color). **(B)** XRD OF magnetite nanoparticles.

**Figure 2:**
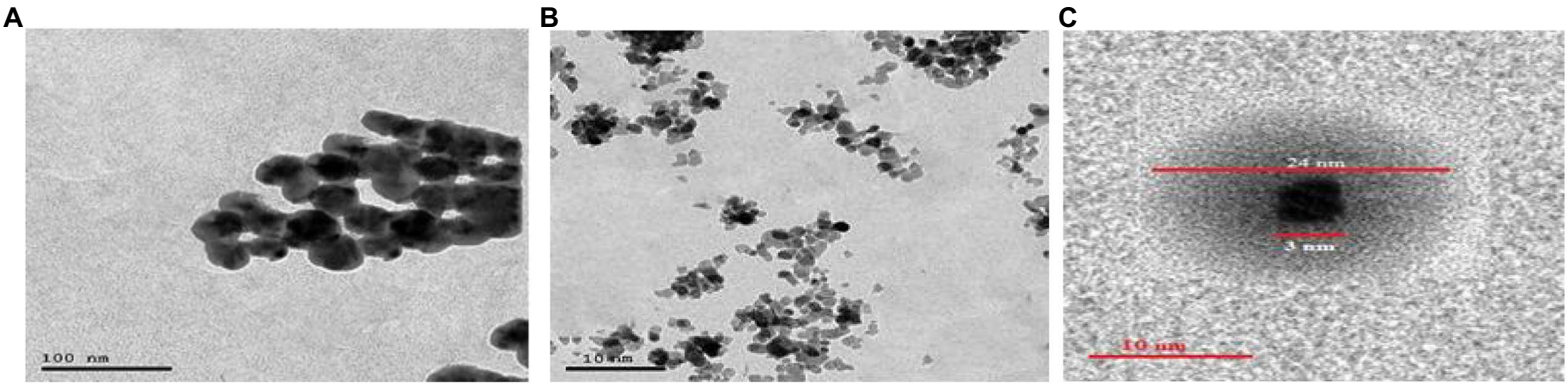
**(A)** Transmission electron microscopy (TEM) of silver nanoparticles. **(B)** Magnetite nanoparticle. **(C)** Silver magnetite core shell nanoparticle.

**Figure 3:**
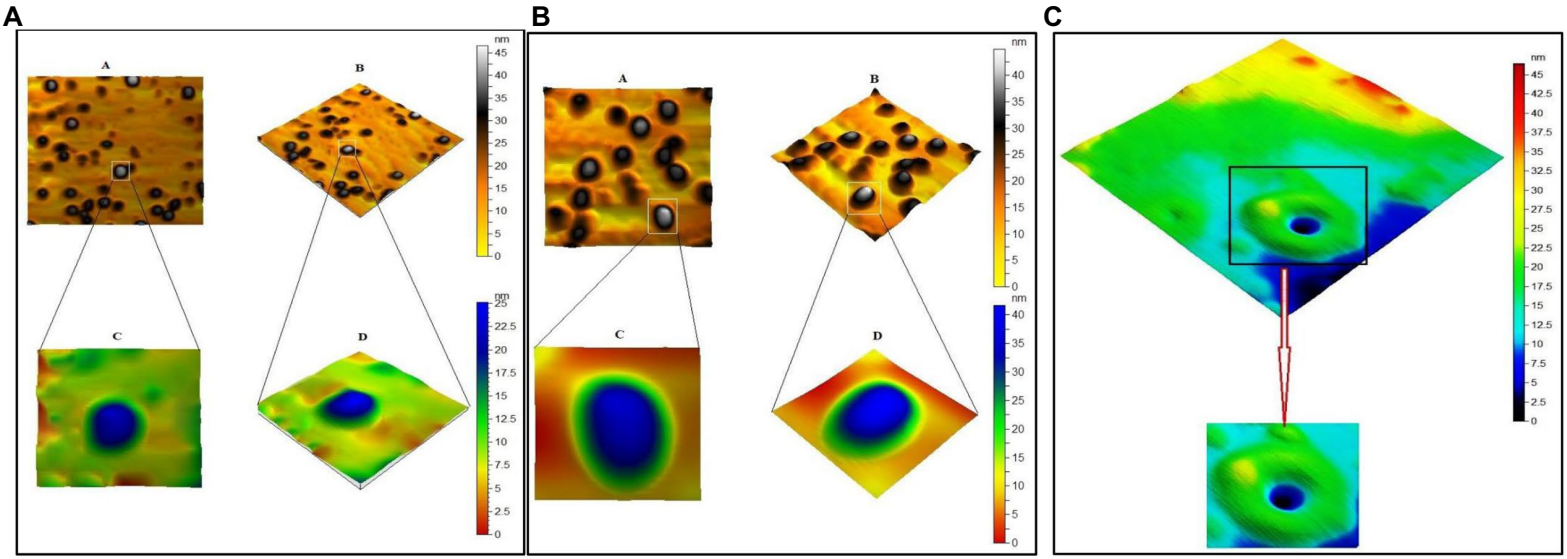
**(A)** Atomic Force Microscope (AFM) of silver nanoparticles. **(B)** Magnetite nanoparticle. **(C)** Silver magnetite core shell nanoparticle.

The crystallinity of silver nanoparticles, magnetite nanoparticles, and silver/magnetite core shells was investigated by X-ray diffraction (XRD). Figure 1 (blue color) displays an X-ray diffraction pattern for silver nanoparticles with peaks at 2 = 35.634, 43.415, and 65.264°, which correspond to 111, 220, and 400, respectively. According to the Joint Committee on Powder Diffraction standards (JCPDS-4-0783 Diff. card), it was matched with the standard silver diffraction pattern. The Plain magnetite XRD pattern, as shown in Figure 2, has peaks at 30.344, 35.634, 43.415, 57.264, and 62.866°, which correspond to 220, 311, 222, 400, and 511, respectively, and is consistent with the standard magnetite diffraction pattern of Fe_3_O_4_ as defined by the Joint Committee on Powder Diffraction standards (JCPDS 19–0629 Diff. card.). As shown in Figure 1 with red color, silver/magnetite nanocomposite has a pattern of 35.634, 37.25, 43.415, 57.264, 62.866, 65.26, and 77.5°, which are assigned to 111, 220, 222, 311, 400, and 511. It refers to the fact that Ag only covers a thin layer of Fe_3_O_4_ and that some magnetite particles remain bare. The presence of a core shell structure magnetite core with a silver shell was confirmed by XRD data (Gong et al., 2007). Dynamic Light Scattering (DLS) data revealed that the diameters of silver nanoparticles, magnetite, and silver/magnetite shell core nanoparticles were 25, 30, and 25 nm, respectively. The zeta potentials were −15, −10.3, and −9 mv for silver nanoparticles, magnetite, and silver/magnetite shell core nanoparticles, respectively. The data indicated that a thin layer of silver nanoparticles may be covered around a magnetite core. TEM and AFM were applied to identify the morphology and forms of Ag, Fe_3_O_4_, and Ag@Fe_3_O_4_ -NPs. Figures 2A, 3A show that silver nanoparticles have spherical shapes with diameters in the range of 30–35 nm, whereas magnetite nanoparticles have a polygonal shape with diameters in the range of 25–30 nm (Figures 2B, 3B). Ag@Fe_3_O_4_ -NPs has a cubic core with a diameter of 3 nm surrounded by a silver shell with a diameter of 25 nm, which emphasizes the production of silver magnetite core shell nanoparticles with regular morphologies and without aggregation, as shown in Figures 2C, 3C.

### The antibacterial activity

The antibacterial activity of the testing material (Ag-NPs, Fe_3_O_4_ NPs, and Ag@ Fe_3_O_4_ -NPs) was investigated against *S. typhimurium* and *E. coli.* MIC and MBC of Ag-NPs, Fe_3_O_4_ NPs, and Ag@ Fe_3_O_4_ core shell results are summarized in Tables 1, 2. All of the materials examined had antimicrobial activity. The results showed that Ag@ Fe_3_O4 is more effective than Ag-NPs and Fe_3_O_4_ NPs at inhibiting selected Gram–negative foodborne pathogens, *S. typhimurium* and *E. coli*. MIC value of Ag@Fe_3_O_4_ -NPs core shell against foodborne pathogens was 3.1 μg/ml for *S. typhimurium* and 5.4 μg/ml for *E.coil*, whereas Ag-NPs and Fe_3_O_4_-NPs values for *S. typhimurium* were 4.1 and 6.9 μg/ml, respectively. Ag-NPs and Fe_3_O_4_-NPs had MICs of 8.2 and 10.3 μg/ml against *E.coil*, respectively. MIC values of Polymyxin and Kanamycin against *E. coli* and *S. typhimurium* were 4.5 and 2.5, respectively.

**Table 1:** The diameter of zone inhibition (mm), MIC value (mg/ml), and MBC value (μg/ml) for *Escherichia coli.*

Test material	Diameter of inhibition zone (mm)	MIC (μg/ml)	MBC (μg/ml)
Ag-NPs	20 mm	8.2 ± 0.02^*^	13.4 ± 0.36^*^
Fe_3_O_4_-NPs	15 mm	10.3 ± 0.13^*^	16.7 ± 0.05^*^
Ag@Fe3O4 -NPs core shell	28 mm	5.4 ± 0.12	7.9 ± 0.01^*^
Polymyxin	33 mm	4.5 ± 0.25	6.8 ± 0.01^**^

* *p* < 0.05 was considered a statistically significant difference.

** *p* < 0.001 was considered a statistically significant difference.

**Table 2:** The diameter of zone inhibition (mm), MIC value (mg/ml), and MBC value (μg/ml) for *Salmonella typhimurium.*

Test material	Diameter of inhibition zone (mm)	MIC (μg/ml)	MBC (μg/ml)
Ag-NPs	22 mm	4.1 ± 0.15*	6.3 ± 0.1^*^
Fe_3_O_4_-NPs	17 mm	6.9 ± 0.3*	8.7 ± 0.2^*^
Ag@Fe_3_O_4_ -NPs	30 mm	3.1 ± 0.2	5.4 ± 0.01^**^
Kanamycin	37 mm	2.5 ± 0.1	4.2 ± 0.01**

* *p* < 0.05 was considered a statistically significant difference.

** *p* < 0.001 was considered a statistically significant difference.

The minimum bactericidal concentration is defined as the lowest antibacterial agent concentration required to kill bacteria. Ag@Fe_3_O_4_ -NPs core shell nanoparticles had MBCs of 5.4 and 7.9 μg/ml against *S. typhimurium* and *E. coil*, respectively. MBC values for silver nanoparticles and magnetite against *E. coil* were 8.7 and 6.3 μg/ml*, respectively.* In this study, the MBC values for silver nanoparticles and magnetite against *S. typhimurium* were 13.4 and 16.7 μg/ml, respectively. MBC values of Ag-NPs and Fe_3_O_4_-NPs against *E. coil* were 8.2 and 10.3 μg/ml, respectively. MBC values for polymyxin and kanamycin against *E*. *coil and S. typhimurium were 4.5* and 2.5, respectively.

#### Colony forming unit measurement

**Figure 4:**
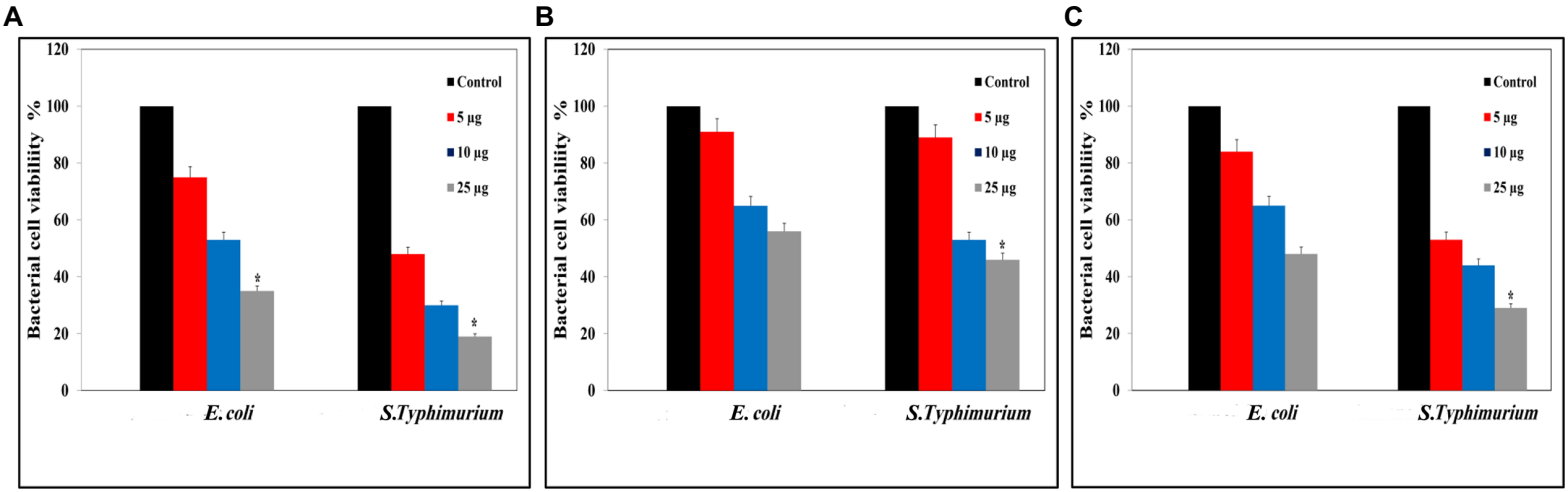
Colony Forming Unit (CFU) assays: **(A)** CFU of Silver magnetite core shell nanoparticle against *Escherichia coli* and *Salmonella typhimurium.* **(B)** CFU measurements of magnetite nanoparticle against *E. coli* and *S. typhimurium*. **(C)** CFU measurements of silver nanoparticle against *E. coli* and *S. typhimurium*. ^*^p < 0.05 was considered a statistically significant difference.

Colony forming unit data displayed those high concentrations of Ag@Fe_3_O_4_ -NPs core shell nanoparticles have a bactericidal activity against *S. typhimurium* and *E. coil,* as Figure 4A showed. Ag@Fe_3_O_4_ -NPs core shell nanoparticles reduced the viability of *S. typhimurium* and *E. coli* to less than 20 and 30, respectively. However, magnetite nanoparticles reduced the viability of *S. typhimurium* and *E. coli* to 50 and 45%, respectively, as Figure 4B revealed. Silver nanoparticles have a good effect on the viability of both bacteria cells. As Figure 4C displays, it reduced the viability of *S. typhimurium* and *E. coli* to 45 and 35%, respectively.

The results show a strong antibacterial propensity of Ag@Fe_3_O_4_ -NPs against studied bacterial strains. Similarly, the data support the antibacterial growth kinetic study of all testing materials.

#### Time-kill assay

**Figure 5:**
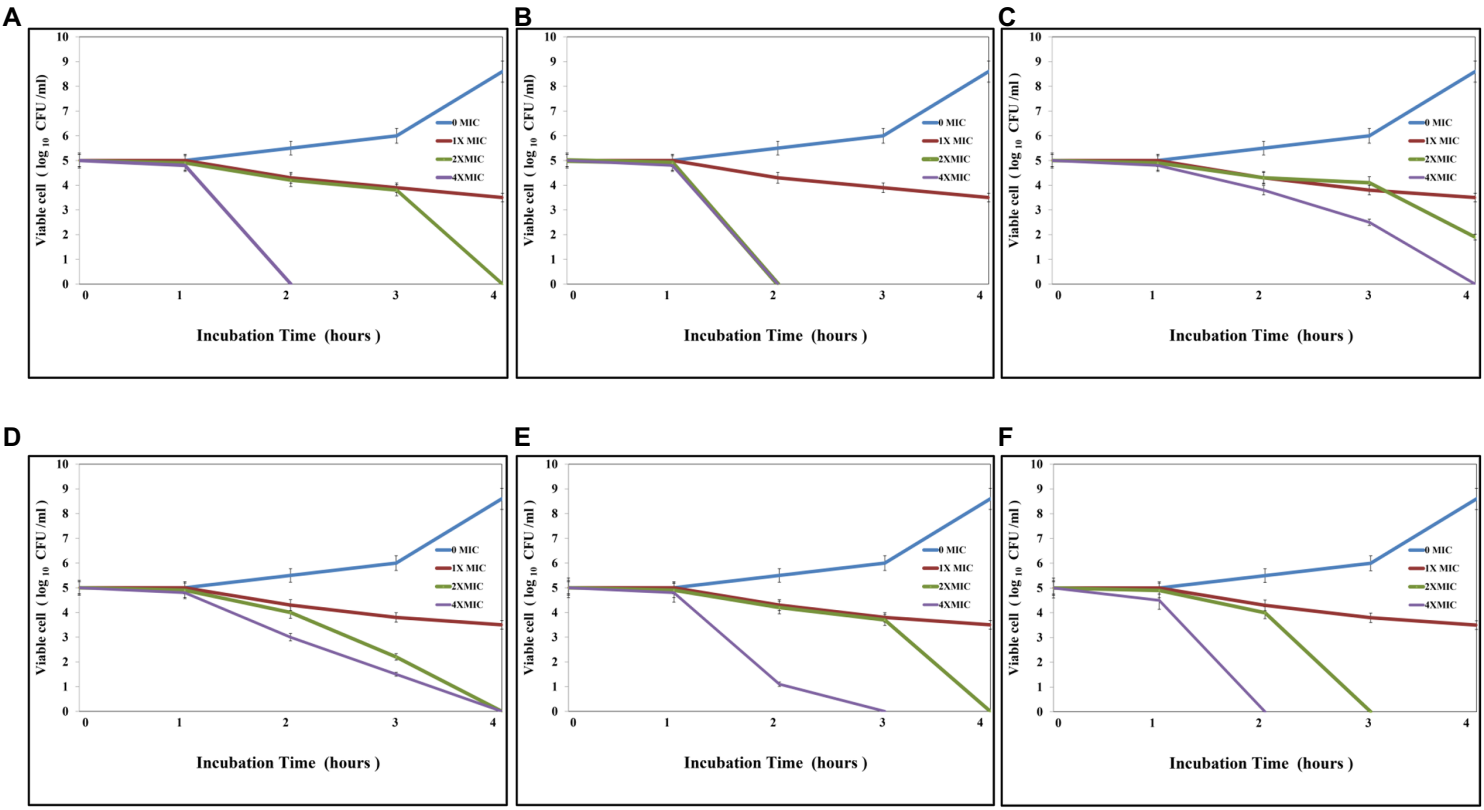
Kill time assays: **(A)** Silver magnetite core shell nanoparticle against *Escherichia coli*. **(B)** Silver magnetite core shell nanoparticles against *Salmonella typhimurium*. **(C)** Magnetite nanoparticles against *E. coli*. **(D)** Magnetite nanoparticles against *S. typhimurium*. **(E)** Silver nanoparticles against *E. coli*. **(F)** Silver nanoparticles against *S. typhimurium.*

As Figure 5 shows, the antibacterial activity of Ag@Fe_3_O_4_ -NPs was effective against *E. coli,* which decreased the number of CFU/mL by 3 log units (99%). As shown in Figure 5A, the time kill of Ag@Fe_3_O_4_ -NPs against *E. coli* was achieved after 2 h of incubation at 2xMIC (10.8 μg/ml) and 4xMIC (21.6 μg/ml), respectively. Figures 5C,E show that after 2 h of incubation at 4XMIC, Ag-NPs and Fe_3_O_4_ were less effective for *E. coli* (16.4 and 20.6 μg/ml, respectively). Also, as Figure 5B revealed, the bactericidal activity of Ag@Fe_3_O_4_ -NPs was effective against *S. typhimurium.* The killing kinetic time of Ag@Fe_3_O_4_ -NPs against *S. typhimurium was* reached after 2 h of incubation at 2xMIC (6.2 μg/ml) and 4xMIC (12.4 μg/ml) while Ag-NPs and Fe_3_O_4_ were less effective for *E. coli* after 2 h of incubation at 2xMIC (8.2 and 13.8 μg/ml) As shown in Figures 5D,F, 4X MIC (16.4 and 27.6 μg/ml) was used to extract the samples. This suggests that Ag@Fe_3_O_4_ -NPs core shell nanoparticles are effective against Gram-negative bacteria strains.

#### Flow cytometry analysis

**Figure 6:**
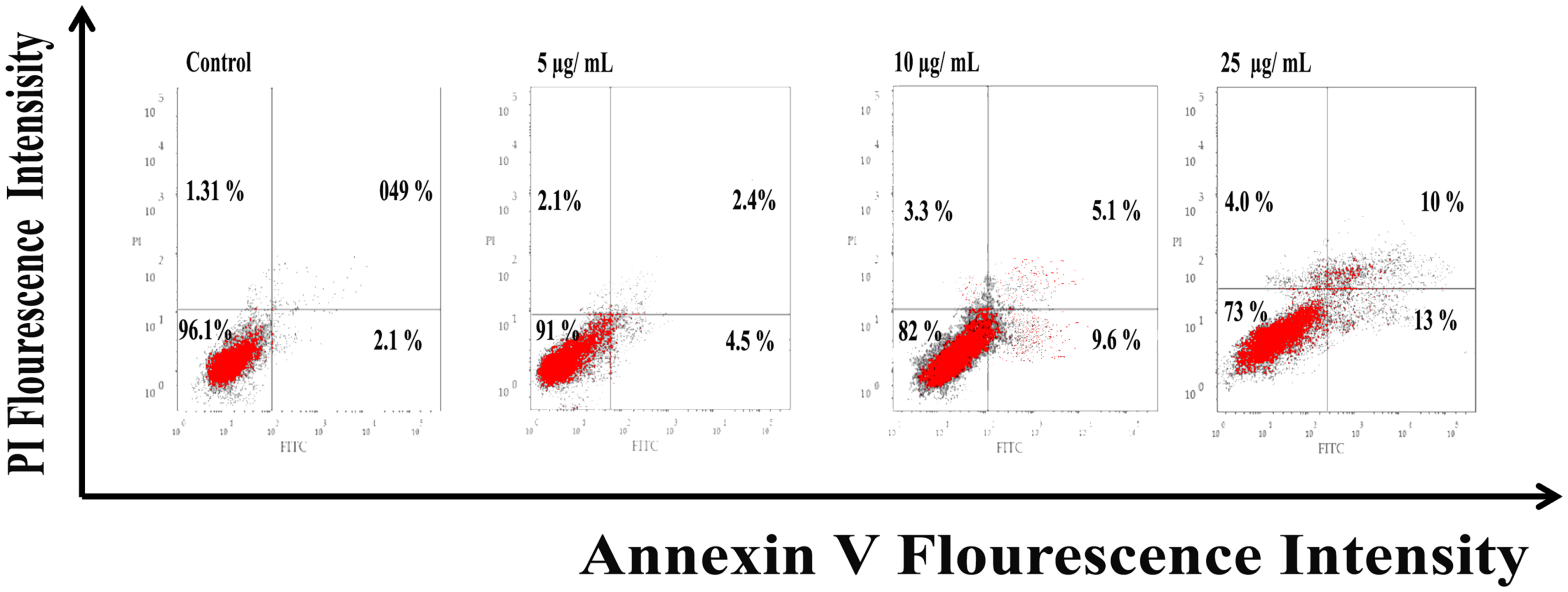
Flow cytometric analysis of silver magnetite core shell nanoparticles induced apoptosis against *Escherichia coli* cells.

**Figure 7:**
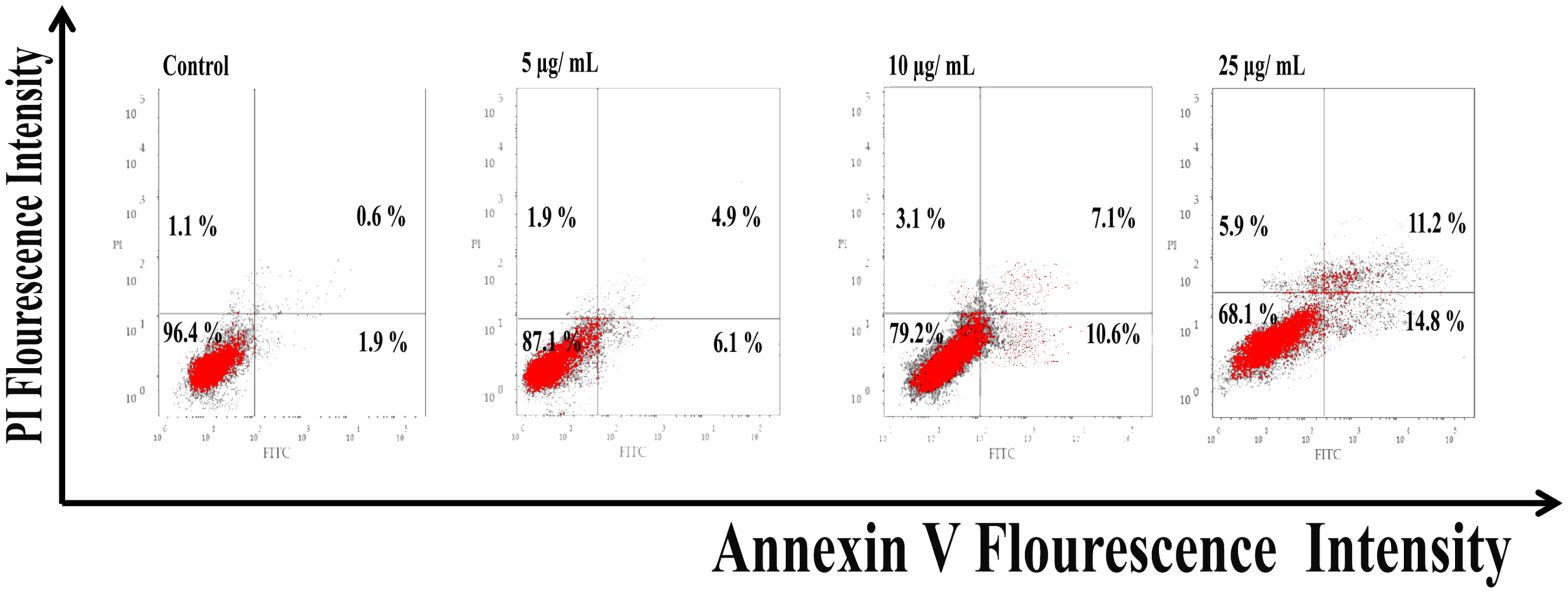
Flow cytometric analysis of silver magnetite core shell nanoparticles induced apoptosis against *Salmonella typhimurium.*

As shown in Figure 6, Ag@Fe_3_O_4_ -NPs induced apoptosis in *E. coli* cells using Annexin V-FITC/PI staining. The percentage of apoptotic cells (including early and late apoptotic cells) increased with increasing concentrations of silver magnetite core shell nanoparticles, rising from 6.9% at 5 μg/ml to 23% at 25 μg/ml. As shown in Figure 7, the percentage of apoptotic cells (including early and late apoptotic cells) increased with an increasing concentration of silver magnetite core shell nanoparticles, from 11% at 5 μg/ml to 26% at 25 μg/ml, respectively.

#### Reactive oxygen species

**Figure 8:**
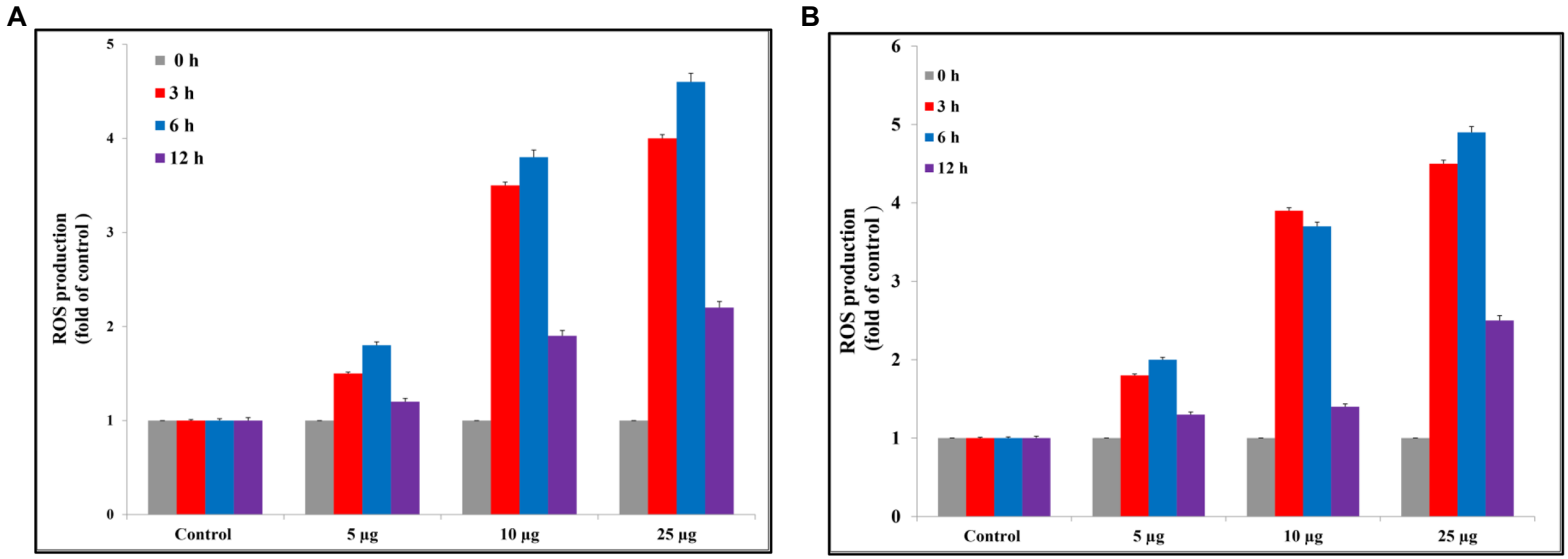
Reactive oxygen species assay. **(A)** Silver magnetite core shell nanoparticle against *Escherichia coli.* **(B)** Silver magnetite core shell nanoparticle against *Salmonella typhimurium.*

Reactive oxygen species are a major factor that causes apoptosis in bacteria. Ag@Fe_3_O_4_ -NPs induces the generation of ROS that causes the killing of *S*. *typhimurium* As Figure 8A showed, Ag@Fe_3_O_4_ -NPs increased the production of ROS from 1.8, 1.5, and 1.4 at concentrations of 5 μg/ml (3, 6, and 12 h, respectively) to 4, 4.8, and 2.5 after (3, 6, and 12 h, respectively) at concentrations of 25 μg/ml. Also, Ag@Fe_3_O_4_ -NPs induces the generation of ROS that causes the killing of *E. coli* cells As Figure 8B showed, Ag@Fe_3_O_4_ -NPs increased the production of ROS from 1.7, 2, and 1.25 at concentrations of 5 μg/ml (3, 6, and 12 h, respectively) to 4.7, 5, and 2.75 after (3, 6, and 12 h, respectively) at concentrations of 25 μg/ml. the data indicated that a dose-dependent increase in ROS generation in *S. typhimurium* and *E. coli* cells.

## Discussion

Recently, foodborne pathogens are considered a major problem, causing food spoilage all around the world. Food contamination can occur during the food processing steps in the food industry, either by direct or cross contamination (Scallan et al., 2011; Xu and Sun, 2013). There are two sources of contamination: during the industrial process by washing water or soiled worker hands or irrigation water, animals, or pathogens exiting the soil. *Escherichia coli* and *Salmonella* are the most common foodborne pathogens that cause diseases. According to the US Centers for Disease Control and Prevention (CDC) data, there are millions of people suffering from diseases in the United States due to foodborne pathogens (McNicholl et al., 2007). Antibiotic activity is limited due to the formation of biofilm on the surface. The formation of bio-film on the surface is due to the adherence of bacteria on the surface by van der Waals, hydrogen bonds, and electrostatic forces. Noble nanometal is a major common anti-bacterial drug due to its unique features such as small size, wide surface area, and toxicity against many bacteria strains (Bush et al., 2011).

The interaction of metal-based nanoparticles with bacterial envelopes results in good bactericide performance and penetrates bacterial cell walls and membranes, resulting in bacteriostatic and bactericidal effects (Ma et al., 2022). The application of metal-based nanoparticles as antibacterial agents is a new approach to minimize microbial resistance and toxicity to human cells (Alabresm et al., 2021).

Silver nanoparticles are considered an active antimicrobial agent due to their ability to liberate silver ions that kill microbial cells. Also, magnetite nanoparticles are considered a good anti-bacterial agent due to their excellent properties, such as biocompatibility, chemical stability, and antimicrobial activity. Ag@Fe_3_O_4_ -NPs combine the benefits of both silver and magnetite nanoparticles and can be used in the medical field.

The combination of MNPs and silver nanoparticles improves the silver’s biocompatibility and eliminates its contact with the cells. Ag@Fe_3_O_4_ -NPs has a higher bactericidal efficiency than Ag-Fe_2_O_3_ heterodimers or simple Ag. Previous studies mentioned the activity of silver/iron oxide nanoparticle compacts and their antibacterial properties. Bi-functional Fe_3_O_4_@Ag nanoparticles were synthesized by utilizing the water-in-oil microemulsion approach to reduce AgNO_3_ on the surface of Fe_3_O_4_ nanoparticles. The bi-functional Fe_3_O_4_@Ag nanoparticles were approximately 20 nm in size (Gabrielyan et al., 2020). Also, silver nanoparticles synthesized by an electrochemical method and citric acid-coated iron oxide against *E. coli* wild type and *S. typhimurium* MDC1759, comparing the antibacterial activity of Ag-NPs and Fe_3_O_4_ nanoparticle coating with citric acid (Alabresm et al., 2021). Also, citric acid has antibacterial efficiency (Padilla-Cruz et al., 2021).

**Figure 9:**
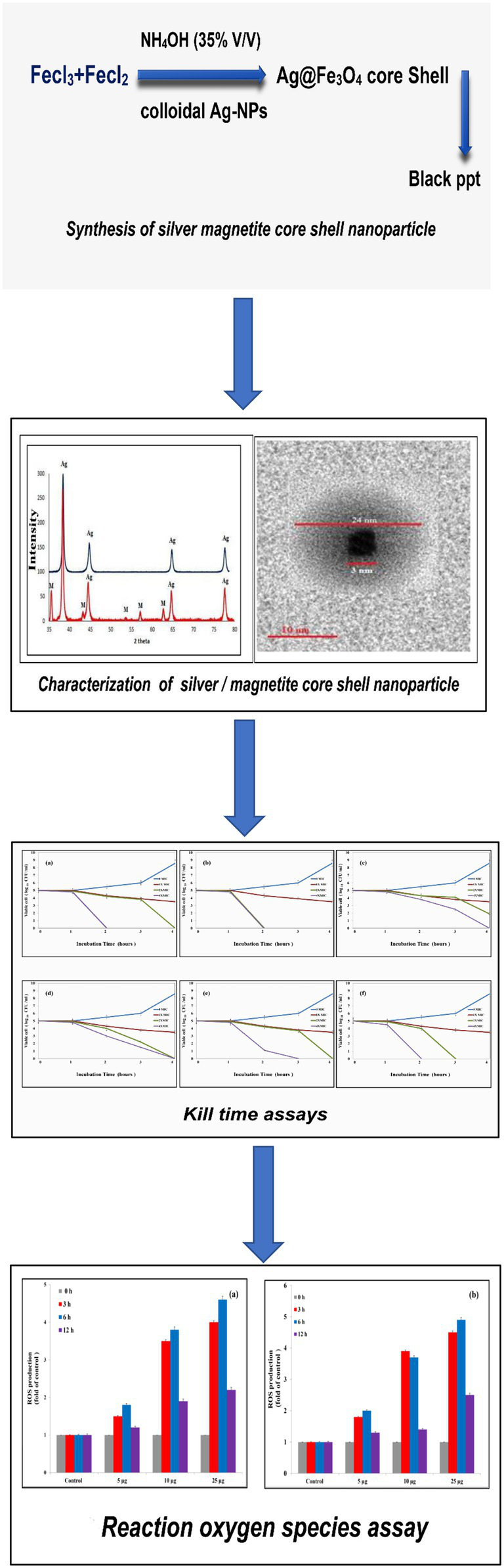
Schematic figure of antibacterial activity of Ag@Fe_3_O_4_ -NPs core shell against Gram-negative foodborne pathogens.

Padilla et al. reported that Ag-Fe bimetallic nanoparticles were synthesized by using an aqueous extract from the leaves of *Gardenia jasminoides*. In this study, as Figure 9 displayed the schematic steps of this study. Firstly, Ag@Fe_3_O_4_ -NPs were prepared by using an efficient, economical, and environmentally friendly method. The method depends on co-precipitation of the Fe^+2^/Fe^+3^ with molar ratios of 2/3 by using ammonia solution instead of NaOH in the absence of nitrogen gas (Mascolo et al., 2013; Hassan et al., 2019). The function of an ammonia solution is to combine with H_2_O and dissociate into ammonia gas and hydroxide ions, which then precipitate magnetite. Whenever ammonia gas gives nitrogen to the atmosphere; it prevents oxygen from oxidizing iron salts into hematite (Lara et al., 2010). Ag@Fe_3_O_4_ -NPs a magnetite core nanoparticle surrounded by a silver cloud with a controlled shape, and investigated its antibacterial activity against the most common foodborne pathogens. Silver magnetite core shell nanoparticles have a cubic core with a diameter of 3 nm surrounded by a silver shell with a diameter of 25 nm, which emphasizes the formation of silver magnetite core shell nanoparticles with regular morphologies and without any aggregates. Secondly, the bacterial activity was evaluated using the Resazurin dye (Michels et al., 2017). The results showed that Ag@Fe_3_O_4_ -NPs were more effective than magnetite and silver nanoparticles against *E. coli* and *S. typhimurium.* The results showed that *E. coli* was less vulnerable to all of the testing materials than *S. typhimurium* due to the lipopolysaccharide blockage (Michels et al., 2017). In addition, the kill time assay revealed that Ag@Fe_3_O_4_ -NPs nanoparticles are more effective than Ag-NPs and MNPs against Gram-negative bacteria strains.

The antibacterial mechanism of Ag@Fe_3_O_4_ -NPs is based on two methods. The first is based on the action of free silver ions. The cell membrane is a built-up of protein and lipids. Silver ions can interact with proteins to form complexes with molecules containing oxygen, phosphorus, sulfur, or nitrogen atoms, which inactivate the membrane-bound proteins and enzymes. Previous studies reported that silver particles can bind to unsaturated fatty acids on the cell membrane and alter their dynamic function, which alters membrane permeability and integrity (Piao et al., 2011). Michels et al. mentioned that nanomaterials such as silver and magnetite nanoparticles investigated against ammonia-oxidizing bacteria adsorb continuously on the outer surface of the microbial cell membrane until the morphology of the surface layer is entirely destroyed (Michels et al., 2017). The amorphous thin porous oxide shell of Ag@Fe_3_O_4_ -NPs makes it easier to liberate silver ions from the outer silver surface, particularly Ag-NPs (Dakal et al., 2016).

The antibacterial activity of silver nanoparticles depends on the shape and size of the nanoparticles. Smaller sizes of silver nanoparticles within the size range of 5–10 nm can cause cell membrane damage and degradation of the bacterial chromosome (Alavi and Rai, 2021). Additionally, ROS is considered a major mechanism of toxicity *in vitro* and *vivo* generated by metal-based NPs such as noble metals and metal oxide NPs (Alavi et al., 2022). For instance, CuO and Ag-CuO-NPs in a spherical shape with a diameter of 18 and 20 nm, inhibited *S. aureus* with inhibition zones of 19 and 15 mm, respectively. Moreover, cleavage of pBR 322 DNA was observed at high levels for CuO-NPs relative to Ag-CuO NPs (Jadhav et al., 2018).

Interestingly, silver nanoparticles can penetrate the bacterial cell wall, where they contribute to the creation of free radicals and the generation of ROS, which leads to cell death. Similarly, silver ions released from nanoparticles into the cytoplasm might cause oxidative stress, causing dispersion in membrane morphology. As a result, silver can alter cellular architecture and intracellular biological processes, ultimately leading to death (Auffan et al., 2009). Iron may also interact with particular amino acids (–SH groups of cysteine) that are found in bacterial cell wall proteins (Auffan et al., 2009). Piao et al. reported that the breakdown of the mitochondrial membrane is the primary cause of apoptosis (Piao et al., 2011). Our data showed that after 12 h of exposure to Ag@Fe_3_O_4_ -NPs, apoptosis increased in a concentration-dependent manner. As shown in Equations 2, 3, ROS production is followed by the Fenton reaction, or the Haber-Weiss cycle, which accelerates the synthesis of H_2_O_2_ and causes DNA and protein damage. Also, as shown in Equations 1, 2, the presence of the magnetite species Fe^+2^ and Fe^+3^ in the culture media activates the formation of ROS according to the Haber-Weiss cycle. According to our data, the formation of reactive oxygen species is concentration and time-dependent.


(1) $$Fe\left(III\right)+H_{2}O_{2}=Fe\left(II\right)+{OH}^{-}+{OH}^{\circ }$$

(2) $$Fe\left(II\right)+H_{2}O_{2}=Fe\left(III\right)+{HO}_{2}+H^{+}$$

## Conclusion

In the present study, Ag/Fe_3_O_4_ core shell NPs, Ag-NPs, and Fe_3_O_4_ NPs were synthesized by wet chemical reduction and co-precipitation methods and characterized by physiochemical techniques such as X-ray diffraction (XRD), Dynamic Light Scattering (DLS), Transmission electron microscopy (TEM), and Atomic Force Microscopy (AFM). The antibacterial efficacy of all the testing materials was evaluated by using the Kirby–Bauer Disk Diffusion test, MIC, MBC, CFU, and Killing Kinetics assays against *S. typhimurium* and *E. coli,* which were chosen as Gram-negative foodborne pathogens. Furthermore, the cytotoxicity mechanism of Ag/Fe_3_O_4_ core shell NPs was investigated using flow cytometer analysis and apoptosis and ROS assays. The results showed that Ag/Fe_3_O_4_ core shell NPs with a diameter of 3 nm were synthesized with a cubic core surrounded by a silver shell of 25 nm, confirming the production of silver magnetite core shells with regular morphologies and no aggregation. MIC values for Ag/Fe_3_O_4_ core shell NPs against *S. typhimurium* and *E. coli* were 3.1 and 5.4 μg/ml, respectively, whereas the MIC values for Ag-NPs and MNPs against *S. typhimurium* and *E. coli* were 4.1, 8.2 μg/ml for Ag-NPs and 6.9, 10.3 μg/ml for MNPs. CFU measurements revealed that Ag/Fe_3_O_4_ core shell NPs reduced *S. typhimurium* and *E. coli* viability by 19 and 35%, respectively. Also, the kill time test indicated that Ag/Fe3O4 core shell NPs reached a number of CFU/mL of 3 log units (99 percent) to zero value at 2xMIC (10.8 μg/ml) and 4xMIC (21.6 μg/ml) after 2 h of treatment with testing materials. Furthermore, the data revealed that the Ag@Fe_3_O_4_-NPs core shell nanoparticles’ ability to induce apoptosis and ROS in *S. typhimurium* and *E. coli* was concentration and time dependent. The antibacterial activity of silver nanoparticles depends on the shape and size of the nanoparticles. Smaller sizes of silver nanoparticles within the size range of 5–10 nm can cause cell membrane damage and degradation of the bacterial chromosome. In addition, ROS is considered a major mechanism of toxicity *in vitro* and vivo when generated by metal-based NPs such as noble metals and metal oxide NPs such as Fe_3_O_4_-NPs. The capacity of Ag@Fe_3_O_4_ -NPs core shell nanoparticles to release free silver ions and form reactive oxygen species *via* the Fenton reaction, or Haber-Weiss cycle, was shown to be a plausible mechanism for explaining the toxicity of silver/magnetite core shell nanoparticles. Finally, the compact Ag@Fe_3_O_4_-NPs core shell nanoparticles exhibit higher antibacterial activity than silver nanoparticles and magnetite nanoparticles against Gram-negative foodborne pathogens.

## Data availability statement

The original contributions presented in the study are included in the article/supplementary material, further inquiries can be directed to the corresponding author.

## Author contributions

ES: conceptualization and supervision. ES and AH: formal analysis, validation, visualization, and writing-original draft. FA-S, EF, and ES: funding acquisition. AH, FA-S, and ES: investigation. ES, FA-S, AH, and EF: methodology. ES and EF: project administration. FA, EF, and ES: resource. AH, DD, and FA: writing—review edition. All authors contributed to the article and approved the submitted version.

## Funding

This work was fully supported by Taif University Researchers supporting project number (TURSP-2020/113), Taif University, Saudi Arabia.

## Conflict of interest

The authors declare that the research was conducted in the absence of any commercial or financial relationships that could be construed as a potential conflict of interest.

## Publisher’s note

All claims expressed in this article are solely those of the authors and do not necessarily represent those of their affiliated organizations, or those of the publisher, the editors and the reviewers. Any product that may be evaluated in this article, or claim that may be made by its manufacturer, is not guaranteed or endorsed by the publisher.
